# Inflammatory fibro-epithelial hyperplasia related to a fixed implant-supported prosthesis: A case report

**DOI:** 10.4317/jced.54921

**Published:** 2018-09-01

**Authors:** Alba Sánchez-Torres, Inês Mota, Javier Alberdi-Navarro, Iñaki Cercadillo-Ibarguren, Rui Figueiredo, Eduard Valmaseda-Castellón

**Affiliations:** 1 DDS, MS, Master of Oral Surgery and Implantology. Associate Professor of Oral Surgery, School of Medicine and Health Sciences, University of Barcelona. Researcher at the IDIBELL Institute. Barcelona, Spain; 2 DDS, Fellow of Master of Oral Surgery and Implantology. School of Medicine and Health Sciences, University of Barcelona, Spain; 3 DDS, MS, PhD, Oral Medicine and Oral and Maxillofacial Pathology Units, Dental Clinic Service. Department of Stomatology II. University of the Basque Country (UPV/EHU); 4 DDS, MS, PhD, Master of Oral Surgery and Implantology. Associate Professor of Oral Surgery, School of Medicine and Health Sciences, University of Barcelona, Barcelona. Researcher at the IDIBELL Institute. Barcelona, Spain; 5 DDS, MS, PhD, EBOS. Professor of Oral Surgery, Professor of the Master of Oral Surgery and Implantology. School of Medicine and Health Sciences, University of Barcelona. Researcher at the IDIBELL Institute. Barcelona, Spain

## Abstract

The gingival overgrowth is a common finding in the clinical practice with a diverse etiology. There are no treatment guidelines defined for this oral lesions. These can provoke discomfort to the patient and often, can alter the function of the stomatologic system. This article presents a case report of a bilateral gingival overgrowth in a 68 years old woman wearing a fixed upper-arch implant-supported prosthesis placed five years ago. The clinical exam after removing the prosthesis showed an intense accumulation of plaque around the intermediate abutments associated to a mucosal enlargement with suppuration on touching the buccal area of the implant in position 1.5 and a probing depth of 8mm. The 2.4 and 2.5 implants also showed vestibular mucosal enlargement and a probing depth of 6mm. No changes were observed in the peri-implant bone level measured in the periapical radiographs. An incisional biopsy was made on second quadrant and sent for the histopathological study. The definitive diagnosis was inflammatory fibro-epithelial hyperplasia. No recurrence has been reported after a 6 month follow-up.

** Key words:**Fibro-epithelial hyperplasia, gingival enlargement, gingival overgrowth, full-arch implant-supported prosthesis.

## Introduction

Gingival reactive lesions are one of the main pathologies that affects the gingival tissue (1). Within these lesions are included the inflammatory fibrous hyperplasia, pyogenic granuloma, peripheral giant cells granuloma and peripheral ossifying fibroma, with specific clinicopathological characteristics (2). The histological changes in the mucosal tissues have been identified as hypertrophy (an increase in the size of the cellular elements making up the gingivae) or hyperplasia (an increase in the number of the cellular elements) (3).

Nowadays, in the clinical practice, the term “gingival hyperplasia” is used based on the clinical appearance rather than histological evidence. Thus, it would be more appropriate for clinicians to use the clinical term “gingival enlargement” in the absence of histological confirmation (3).

The term inflammatory hyperplasia is used to describe a large range of common occurring nodular growths of the oral mucosa that histologically represent inflamed fibrous and granulation tissue. The size of these reactive hyperplastic masses is variable, depending on the intensity and type of irritant stimulus and besides, on the inflammation degree of the affected tissue.

The main etiological factor seems to be the chronic trauma due to poorly fitting dental prostheses, calculus, over-contoured restorations, acute or chronic lesions due to bites or fractured teeth (4) as well as a poor plaque control that results in mucosal irritation, inflammation and proliferation (3-5). The presence of this type of reactive lesions in peri-implant mucosa has been poorly described and there is some controversy about clinicopathological and etiopathogenic aspects.

The gingival overgrowth, depending on its extension, could have multiple effects on the stomatognatic system: functional disorders (impaired speech), difficulty in chewing and even aesthetic problems that could cause psychological impairment (4).

The aim of this article is to describe the clinicopathological characteristics of a patient with a fixed upper-arch implant supported prosthesis with bilateral gingival enlargement.

## Case Report

**Figure 1:**
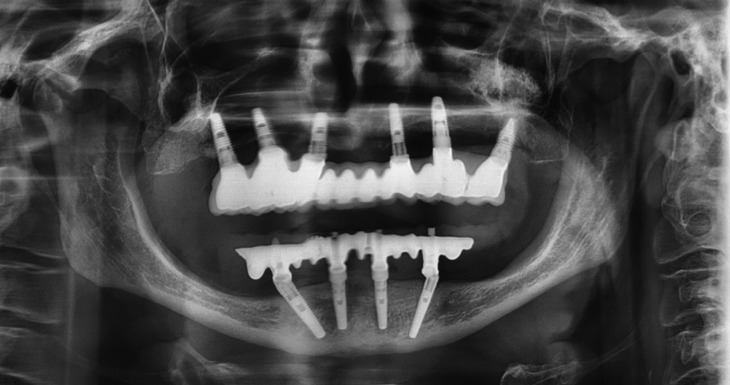
Ortopantomography.

**Figure 2:**
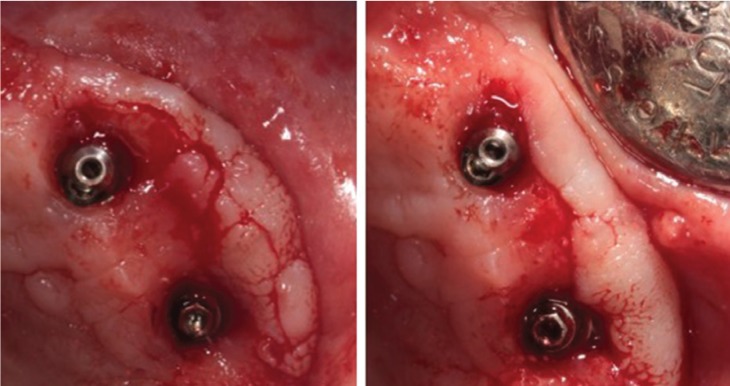
Surgical treatment on the second quadrant.

The patient was a 68 years old woman suffering from depression, hypothyroidism, arrhythmias and hypercholesterolemia, pharmacologically controlled with clomipramine 25mg (0-0-1), lormetazepan 2mg (0-0-0.5), fluoxetine 20mg (1-0-0), levotiroxin 100mg (1-0-0), bisoprolol 2.5mg (1-0-0) and simvastatin 20mg (0-0-1). She did not have toxic habits neither allergies. The patient attended the dental clinic because of pain on the right side of the upper jaw. She wore a fixed upper-arch implant supported prosthesis placed five years ago and she had not attended the control visits for the last 2 years (Fig. 1). The clinical exam after removing the prosthesis showed intense accumulation of plaque (both in the prosthesis and in the intermediate abutments) and a mucosal enlargement with suppuration on palpating the vestibular area of the implant in position 1.5 and a probing depth of 8mm. The implants in position 2.4 and 2.5 also showed vestibular mucosal enlargement and a probing depth of 6mm. Periapical radiographs showed no changes on the peri-implant bone level. Therefore, it was decided to perform a surgical treatment of the implant 1.5 under local anesthesia (articaine 4% and epinephrine 1:200.000) with a full-thickness trapezoidal flap. After rising the flap, a correct bone level and the absence of exposed implant threads were observed. Hence, the thickness of the vestibular flap was reduced and the flap was repositioned with 4/0 monofilament suture. On the left side, an incisional biopsy was made in order to reduce the vestibular thickness and send the sample for the histological study (Fig. 2). The presumptive diagnosis was gingival hyperplasia due to plaque accumulation.

The lesion was immersed in a 10% formaldehyde solution and sent to the Oral and Maxillofacial Pathology and Diagnosis Service (SDPOMF) for the histopathological exam.

**Figure 3:**
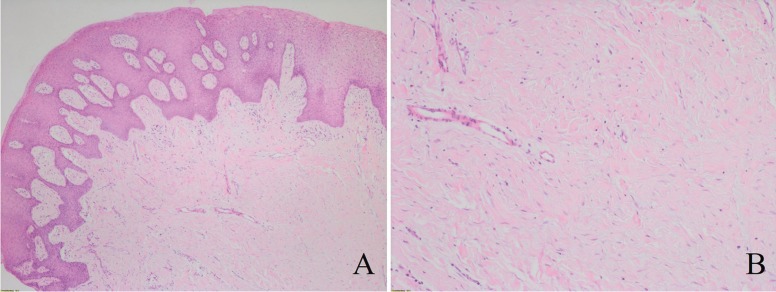
A) Epithelial hyperplasia and connective fibrocellular tissue (H&E 20x). B) Fibrocellular collagen connective tissue with scarce cellularity (H&E 40x).

The histopathological exam found that the lesion was mainly constituted by fibrocellular collagen connective tissue with scarce cellularity and a diffuse and mild lymphoplasmacytic chronic inflammatory infiltration. The superficial mucosal epithelium was parakeratinized and hyperplastic but without any dysplastic phenomena (Fig. 3A,B). Hence, the definitive diagnosis was fibro-epithelial hyperplasia with inflammation. No recurrence has been reported after a 6 month follow-up.

## Discussion

The intimate contact between bone and titanium implants was first demonstrated in 1969, and since then the bone-implant interface has been extensively investigated. However, the study of microflora and peri-implant tissues have almost exclusively been carried out over the last decade (5).

Nowadays, implant-supported restorations constitute a common treatment in dentistry. Nevertheless, short and long-term complications may occur. These can be mechanical, when the damage affects the implant or the restorative components, or biological, when there is a damage on peri-implant tissues (6,7).

According to Berglundh *et al.* (7), biological complications have lower prevalence (40-60%) than mechanical ones (60-80%). However, Papaspyridakos *et al.* (6) reported a non-negligible prevalence of an 11% of patients with inflammation under the prosthesis and even a 26% of gingival enlargements (hypertrophy or hyperplasia) after a 10-year follow-up period. Therefore, gingival enlargement appears to be one of the most prevalent biological complications.

Gingival hyperplasia represents an excessive gum growth. Specifically, the presence of plaque is usually the most common etiologic factor. However, it can also appear in patients with good oral hygiene, so in these cases, other factors could cause local irritation. The biological width invasion, the trauma by brushing or ill-fitting prostheses, or the consumption of drugs such as phenytoin (anticonvulsant), cyclosporine (immunosuppressant) or calcium channel blocking drugs are some examples (4).

The changes observed on histological sections appear in both the epithelium and lamina propria of the gingival tissue. Epithelial hyperplasia produced by the proliferation of the epithelial basal layer cell associated to acanthosis origins the penetration of epithelial cords in lamina propria. The inflammatory process is characterized by an intensive fibroblasts proliferation that suggests these alterations are provoked by the presence of plaque. The connective tissue have a predominance of lymphoplasmacytic mononuclear cells (specific immune response) and macrophages (non-specific response), which indicates the presence of a chronic inflammation. Besides, epithelial surface might present hyperkeratinized or parakeratinized areas due to a tissue protection process (4).

It is noteworthy that there are other factors that might induce gingival overgrowth such as allergic reactions (4,5). Schou *et al.* (5) report a case of a persistent hyperplasia even after the improvement of the oral hygiene and after a gingivectomy. After all, the problem was solved by changing the titanium abutment by gold.

Regardless of its etiology, the treatment of gingival enlargements should firstly consist in a hygienic phase based on professional cleaning and education on oral hygiene, followed by a surgical treatment such as gingivoplasty or gingivectomy since, in many cases, it is not possible to reduce its size despite re-establishing an adequate hygiene. The need to perform the histopathological analysis of the excised tissue in all cases, to confirm the clinical diagnosis, is evident. Although malignant lesions associated to dental implants are rare findings, they can have similar clinical characteristics (8,9).

Finally, it would be very interesting to follow up a cohort of patients with these lesions, since their long-term behavior is still unknown.

## References

[1] Carbone M, Broccoletti R, Gambino A, Carrozzo M, Tanteri C, Calogiuri PL (2012). Clinical and histological features of gingival lesions: A 17-years retrospective analysis in a northern Italian population. Med Oral Patol Oral Cir Bucal.

[2] Giglio Peralles P, Borges Viana AP, Da Rocha Azevedo AL, Ramoa Pires F (2006). Gingival and alveolar hyperplastic reactive lesions: Clinicopathological study of 90 cases. Braz J Oral Sci.

[3] Payne AGT, Solomons YF, Tawse-Smith A, Lownie JF (2001). Inter-abutment and peri-abutment mucosal enlargement with mandibular implant overdentures. Clin Oral Impl Res.

[4] Draghici EC, Craitoiu S, Mercut V, Scrieciu M, Popescu SM, Diaconu OA (2016). Local cause of gingival overgrowth. Clinical and histological study. Rom J Morphol Embryol.

[5] Schou S, Holmstrup P, Hjørting-Hansen E, Lang NP (1992). Plaque-induced marginal tissue reactions of osseointegrated oral implants: A review of the literature. Clin Oral Implants Res.

[6] Papaspyridakos P, Chen CJ, Chuang SK, Weber HP, Gallucci GO (2012). A systematic review of biologic and technical complications with fixed implant rehabilitations for edentulous patients. Int J Oral Maxillofac Implants.

[7] Berglundh T, Persson L, Klinge B (2002). A systematic review of the incidence of biological and technical complications in implant dentistry reported in prospective longitudinal studies of at least 5 years. J Clin Periodontol.

[8] Pinchasov G, Haimov H, Druseikaite M, Pinchasov D, Astramskaite I, Sarikov R (2017). Oral cancer around dental implants appearing in patients with\without a history of oral or systemic malignancy: A systematic review. J Oral Maxillofac Res.

[9] Kaplan I, Zeevi I, Haim Tal H, Rosenfeld E, Chaushu G (2017). Clinicopathologic evaluation of malignancy adjacent to dental implants. Oral Surg Oral Med Oral Pathol Oral Radiol.

